# Establishing forensic DNA databases in Africa

**DOI:** 10.1093/fsr/owae024

**Published:** 2024-04-02

**Authors:** Johannes Hendrik Smith, Juanida Suzette Horne

**Affiliations:** School of Criminal Justice, University of South Africa, Preller Street, Muckleneuk Ridge, Pretoria, South Africa; Department of Police Practice, School of Criminal Justice, College of Law, University of South Africa, Preller Street, Muckleneuk Ridge, Pretoria, South Africa

## Dear editors,

The manuscript titled *Forensic DNA database and criminal investigation in the Sahel Region, a need to update the national security policy?* focuses on the views held in Burkina Faso in the Sahel region on using DNA technology to support criminal investigations. It also includes supplementary data from secondary sources about forensic DNA databases [1].

Zeye et al. [1] highlight that 64.9% of the respondents in their study and a significant number of persons in other countries are aware of the critical role of DNA in solving crime investigations in progress. Moreover, among the 470 participants in that study, 91.7% (431) supported incorporating DNA technology in the country’s criminal justice system. Nevertheless, apprehensions concerned the management, administration, and security of the National Forensic DNA Database (NFDD) in Burkina Faso [1]. These results provide valuable information for policymakers to evaluate the adoption of genetic fingerprinting and an NFDD in Burkina Faso, emphasizing ethical aspects while improving criminal investigations. Notably, respondents to the Zeye et al. [1] study indicated that they supported the establishment of an NFDD in their country for criminal investigations and its function in supporting counterterrorism and organized crime initiatives. We value Zeye et al.’s [1] openness to share the results and viewpoints of their work to start a more extensive discussion about creating an NFDD in the Sahel. Their research is invaluable for scientists, local legislators, and other interested parties actively looking for ways to use forensic DNA databases in law enforcement.

Zeye et al. [1] discuss the concept of “genetic fingerprinting” in their recent article. This term typically refers to DNA profiling techniques, such as those pioneered by Sir Alec Jeffreys, aimed at distinguishing between individuals based on their unique genetic markers. However, it is important to note that modern forensic DNA analysis primarily relies on short tandem repeat analysis for constructing DNA profiles and comparing them in forensic databases internationally. The continued use of the term “fingerprinting” in this context may perpetuate an outdated or misleading perception of the scientific processes involved. Therefore, it is crucial to use more precise terminology that accurately reflects the current state of forensic DNA analysis, ensuring clear and accurate communication within our scientific community.

It will be helpful for Burkina Faso to consider lessons learnt in South Africa. Since its creation in 2015, South Africa’s National Forensic DNA Database (NFDDZA) has demonstrated its value as an investigative tool by producing forensic DNA investigative leads (FILs) that aid in investigating specific crimes. This has positively impacted criminal investigation and increased conviction rates due to the higher number of leads and the linking of perpetrators to crime scene samples [2, 3]. Comparison searches on NFDDZA yielded 20 141 DNA person-to-crime (linking persons on the database) and 9 155 DNA crime-to-crime (forensic) FILs, respectively [3]. Moreover, the DNA database has identified 4 313 serial sexual offenders [3]. The legislation regulating NFDDZA ensured that the Constitutional and human rights of the individual against that of society are balanced. The legislation includes several checks and balances and a penalty clause to protect against the misuse or abuse of DNA samples and DNA analysis. Moreover, NFDDZA is only used according to the intended purpose of law enforcement and the identification of missing persons and unidentified bodies. The legislation created the National Forensic Oversight and Ethical Board (NFOEB) to handle complaints and provide oversight on the compliance by the implementers with the legislation. Although the NFOEB has received several complaints, primarily related to timely service delivery, no complaint of the integrity of abuse of NFDDZA has been received. The checks and balances, as well as penalty clauses, should be more aggressively enforced and be effective.

We believe an NFDD in Burkina Faso will only assist in counterterrorism and organized crime initiatives if physical DNA exhibit material is left at crime scenes. In our experience, forensic DNA databases are not designed to contribute significantly to most contemporary counterterrorism and organized crime initiatives that predominantly centre on digital and cybercrime, as well as destructive mechanisms aimed at eliminating DNA traces left by perpetrators. In exceptional instances, DNA FILs assisted in the investigation of counterterrorism and organized crime initiatives in South Africa [2].

Furthermore, Zeye et al. [1] accurately highlight the advantages of data exchange and forensic DNA sharing between governments, including identifying unidentified remains and missing persons. Undocumented individuals (or illegal immigrants) are entering South Africa from African nations at a noticeable rate. African nations currently share minimal, if any, forensic DNA profiles, in contrast to the European Union. Only until a significant number of African countries perform DNA analysis and create NFDDs will forensic DNA sharing become a reality and assist in cross-border initiatives. As such, the NFDD in South Africa is contributing little to the identification of inexplicable human remains and missing persons among the undocumented population [2].

Zeye et al. [1] advise handing over DNA database management to an independent governmental entity in response to respondents’ grave concern about possible misuse and privacy problems related to the management of the NFDD. Concerns about human rights, privacy, security, access, and other uses of the NFDD outside of its intended uses are all legitimate. It is critical that the legislation adequately addresses the accreditation and regulation of forensic services to have the support of the public and judiciary and the accuracy of forensic findings [4–6]. By establishing the NFOEB, the legislation in South Africa establishing NFDDZA recognizes and only partially addresses some of these concerns [7]. The NFOEB is responsible for overseeing statutory compliance relating to some aspects regarding collection of samples from certain categories of persons and concerning forensic DNA analysis in the State Forensic Science Laboratories, looking into complaints from the public regarding the usage of NFDDZA, and imposing sanctions for abuse or misuse [7]. The NFOEB, however, only has oversight over a part of the forensic DNA profiling system and data storage in databases in South Africa; it does not have oversight over all fields of forensic science and the public sector. The legislation mandates that the authorized officer overseeing DNA analysis at the State Forensic Science Laboratory should develop and recommend quality management standards compliant with the International Organization for Standardization (ISO). However, this requirement does not make it mandatory for accreditation of the laboratory against the ISO 17025 standard. Consequently, within the framework of international best practices, which advocate for forensic DNA laboratories to be accredited against ISO 17025, the South African legislation is inadequate, and the laboratories are not subjected to independent peer review to ensure compliance with these standards. Moreover, the forensic professionals working in the forensic DNA laboratories are not regulated. The absence of regulatory oversight for forensic analysts and independent peer assessment of the laboratory through the accreditation process to evaluate adherence to fundamental ISO requirements may undermine the integrity of specific forensic DNA profiles uploaded to the NFDD.

Moreover, a significant drawback of the legislation is the lack of a requirement to improve integration among stakeholders, particularly state departments, which are essential to the value chain—regulating the subsequent investigation by the detectives (investigators) of the FILs, which are the results of the comparison searches conducted on NFDDZA. Additionally, there is no provision for creating multidisciplinary teams consisting of detectives and judicial officers. Moreover, there is no regulatory guidance for reporting and monitoring the investigation and outcomes of the FILs that are communicated to the detectives.

NFDDZA’s autonomy from law enforcement and DNA testing laboratories was an emotive subject of discussion during the bill-drafting stage. The proposal to locate the NFDD under a different state department, like the Department of Health or the Department of Justice, was turned down following a thorough research tour and comparison with worldwide practices and after gaining public feedback. Subsequently, law enforcement in South Africa is responsible for managing and administrating NFDDZA, and the statute requires it to be independently managed from the forensic DNA testing laboratories [7]. Presently, although the NFDD is administered and managed separately from the Forensic Science Laboratory (FSL) in another component, it still resorts with the same division. The members administrating and managing are not members of the FSL, but in a separate section and component within forensic services. We argue that establishing forensic DNA databases as a separate government agency funded directly by the national treasury would be preferable. This solution would provide true independence from the South African Police Service (SAPS)’s forensic services despite the absence of documented breaches or instances of NFDDZA abuse. This solution would create complete autonomy and independence, free from other state department interference.

The Sydney Declaration (SD) on Forensic Science is a significant document that was debated and published [8]. It outlines fundamental principles and definitions in forensic science, aiming to provide a shared understanding and framework for practitioners worldwide. The declaration emphasizes the importance of accountability, mastery, and a case-based approach in forensic science, particularly in evolving technologies like artificial intelligence. It serves as a guiding document for the forensic science community, promoting standards and best practices to ensure the integrity and reliability of forensic evidence and analysis. We concur with Olckers and Ben Khelil [9] that the SD should serve as a crucial reference point for Africa, providing a foundational understanding of forensic science in a region where specific frameworks are often lacking. It offers a basis for harmonization and consensus-building, crucial for navigating the evolving landscape of forensic techniques, including emerging technologies like artificial intelligence. By promoting accountability and mastery among practitioners, the SD aims to elevate forensic science practice globally, and can also do so in Africa, guiding its development despite technological limitations and unique challenges, ultimately supporting the continent and its forensic community in adopting a scientific approach [9]. The implementation of the SD will also foster confidence in the use and application of forensic science by the public.

We urge other African countries to launch forensic programmes that embed the SD and to facilitate the creation of forensic DNA databases around the continent and the exchange of forensic DNA profiles. Moreover, the legislative framework in South Africa that oversees the creation and management of NFDDZA can act as a model for comparable programmes throughout the continent to customize and improve on it. The lessons learnt and shortcomings of the South African legislation can be used to provide more effective and accountable legislation in Africa. It is critical that intensive broad consultative workshops with interest parties in the value chain, but not limited to, representatives from non-government organizations, libertarians, academia, legal fraternity, scientists, and information technology experts, be held to draft legislation and gain input on implementation of forensic DNA databases. These workshops must be supported with continuous forensic awareness initiatives during the drafting, debating, and implementation of any legislation establishing the forensic DNA database. This cooperative effort can help identify undocumented human remains and successfully combat cross-border criminal activity in Africa.

## Authors' contributions

Johannes Hendrik Smith and Juanida Suzette Horne are contributed to the final text and approved it.

## Compliance with ethical standards

This article does not contain any studies with human participants or animals.

## Disclosure statement

The author reported no potential conflict of interest.

## Funding

No sources of funding to declare.
